# Illness script development in pre-clinical education through case-based clinical reasoning training

**DOI:** 10.5116/ijme.5a5b.24a9

**Published:** 2018-02-09

**Authors:** Yvette Keemink, Eugene J.F.M. Custers, Savannah van Dijk, Olle ten Cate

**Affiliations:** 1 Centre for Research and Development of Education, University Medical Centre Utrecht, The Netherlands

**Keywords:** Clinical reasoning, undergraduate education, illness scripts, case-based reasoning

## Abstract

**Objectives:**

To assess illness script richness and maturity in preclinical students after they attended a specifically structured instructional format, i.e., a case based clinical reasoning (CBCR) course.

**Methods:**

In a within-subject experimental design, medical students who had finished the CBCR course participated in an illness script experiment. In the first session, richness and maturity of students’ illness scripts for diseases discussed during the CBCR course were compared to illness script richness and maturity for similar diseases not included in the course. In the second session, diagnostic performance was tested, to test for differences between CBCR cases and non-CBCR cases. Scores on the CBCR course exam were related to both experimental outcomes.

**Results:**

Thirty-two medical students participated. Illness script richness for CBCR diseases was almost 20% higher than for non-CBCR diseases, on average 14.47 (SD=3.25) versus 12.14 (SD=2.80), respectively (p<0.001). In addition, students provided more information on Enabling Conditions and less on Fault-related aspects of the disease. Diagnostic performance was better for the diseases discussed in the CBCR course, mean score 1.63 (SD=0.32) versus 1.15 (SD=0.29) for non-CBCR diseases (p<0.001). A significant correlation of exam results with recognition of CBCR cases was found (r=0.571, p<0.001), but not with illness script richness (r=–0.006, p=NS).

**Conclusions:**

The CBCR-course fosters early development of clinical reasoning skills by increasing the illness script richness and diagnostic performance of pre-clinical students. However, these results are disease-specific and therefore we cannot conclude that students develop a more general clinical reasoning ability.

## Introduction

Clinical reasoning has been defined as the “inferential processes for collecting and analyzing data and making judgments or decisions about the diagnosis or treatment of patient problems”.^1^ Clinical reasoning training in the pre-clinical phase is considered important,^2^ but poses educational and conceptual challenges, as the development of clinical reasoning ability requires both knowledge and clinical experience.^3^

In the mind of an experienced clinician, diseases are represented as illness scripts.^4^^-^^6^ Script theory describes how information becomes structured in and is retrieved from long-term memory to interpret and predict new information.^7^ The inclusion of patient knowledge enables diagnosticians to quickly activate appropriate illness scripts in a diagnostic context and to use this knowledge to decide on further diagnostic actions.^4^^,^^5^ Illness scripts consist of three components: the Fault (pathophysiological mechanisms), the Enabling Conditions (patient features and contextual factors) and the Consequences, (signs and symptoms).^6^^,^^8^ Custers and colleagues added a fourth component which they considered a ‘bin’ that contained information on course of the disease (if untreated), possible further diagnostic activities, frequency of occurrence, and management.^5^ In the present study, we chose to consider ‘Management’ as a separate component – because this is extensively dealt with in medical education – and to categorize all information that did not fit into one of the four components (Enabling Conditions, Fault, Consequences, or Management) in a fifth category, a bin dubbed ‘Miscellaneous’.

Illness scripts do not appear out of the blue; they develop as a consequence of theoretical knowledge acquisition as well as accumulated experience in a practical context. Preclinical education enables students to build a limited repository of illness scripts, with knowledge mostly centered around the Fault and its Consequences.^5^ Rumelhart and Norman distinguish three forms of learning that, together, adequately describe the development of illness scripts.^9^ The first, most basic form of learning is accretion, which means accumulating new knowledge after appropriate exposure to facts. New data structures are added to the existing memory database. Scripts, however, derive their value from practical applicability; hence, the theoretical knowledge acquired through accretion needs to be tuned to enable use in a practical context. Tuning is the process of functional adaptation of knowledge to the context in which it is used. Finally, after repeated use in a practical context, knowledge becomes fundamentally restructured. Restructuring enables individuals to directly interpret incoming information, such as patient features and symptoms, into illness script terms. Restructuring implies that a clinician can easily infer a probable diagnosis on basis of minimal information.^10^ Less advanced students may very well be able to infer symptoms when provided with a diagnosis but not the other way round. Their repository of illness scripts needs to be tuned and restructured by practical experience before they can achieve this.

Before restructuring can occur, accretion and tuning are the primary learning processes. Early in the medical curriculum students are prepared for practical education in the clinic. Most medical curricula require students to study clinical textbooks and learn facts by heart. There are few examples of explicit approaches to train clinical reasoning in undergraduate students; Schmidt and Mamede distinguish two categories: knowledge-oriented and process-oriented approaches.^11^ There is limited evidence that knowledge-oriented approaches have benefits in improving students’ clinical reasoning, whereas process-oriented approaches are largely ineffective.^11^ This resonates with the Rumelhart and Norman model,^9^ which does not assign a specific role to reasoning per se in the development of complex knowledge structures.

### Course description

One method used to teach clinical reasoning is through Case-Based Clinical Reasoning (CBCR) sessions. Since 1997, University Medical Centre Utrecht offers a CBCR course for first- and second-year medical students, based on a model developed at the University of Amsterdam.^12^ The CBCR course of the second year consists of nine sessions in small groups (12-14 students) that meet every 3 to 4 weeks. Cases are presented in a standard format that reflects the way patients present in a doctor’s office or clinic, even though no real (or simulated) patients are involved. The case starts with a vignette of the patient’s initial presentation, followed by questions and assignments for the students. The case covers all stages of the clinical encounter in their usual sequence (history, physical examination, differential diagnosis, diagnostic testing, and management). As an entire session is devoted to only a single case, there is sufficient room for an extensive discussion of all (educationally) relevant aspects. CBCR sessions are led by two students from the group in turn, in a peer teaching arrangement, and facilitated by a clinician moderator (a final-year medical student, four years the group members’ senior).^13^ Though most of the Bachelor-part of the curriculum deals with basic science, as it consists of integrated blocks, students may already have some knowledge of the diseases discussed in CBCR sessions. In terms of Rumelhart and Norman,^9^ the emphasis is on tuning and restructuring previously acquired knowledge, rather than on accretion of new knowledge.

The assessment of the CBCR course includes a test consisting of cases with multiple questions related to each case. The questions are of extended matching format (where students have to select multiple correct answers from a list of options). This type of question is particularly suited to assess students’ differential diagnostic thinking, their knowledge of multiple physical symptoms, diagnostic tests and treatment modalities, et cetera.^13^^,^^14^

Like its predecessor, the CBCR course has consistently received favorable student evaluations.^13^^,^^15^^,^^16^ However, the effect of a CBCR course on the development of illness scripts has never been investigated. Therefore, the aim of current study was to assess the richness and maturity of illness scripts in students after attending a CBCR course. Richness and maturity are two important aspects of illness scripts; an illness script is rich if it involves an elaborate mental representation that can be used to generate relevant information on all four illness script components. An illness script is mature if it can be activated in a diagnostically relevant context. For this to be possible, the emphasis in the script has to move, with increasing expertise, from Fault-related aspects of the disease (pathophysiological knowledge usually not being very helpful in the early stages of the diagnostic process) to Enabling Conditions and Consequences, which are available early in the consultation and play an important role in activation of appropriate illness scripts.^10^ We know of no other courses that explicitly aim to achieve this form of tuning and restructuring of clinical knowledge. Thus, we investigated two research questions:

      1.   Do students generate richer illness descriptions after being probed with a CBCR diagnosis compared with an (equally common) non-CBCR diagnosis, i.e., a disease not dealt with in the course? And do they provide relatively more information on the Enabling Conditions and Consequences aspect of the illness script, compared with Fault-related information?

      2.   When presented with a CBCR case, are students more likely to generate the correct diagnosis than when presented with an (equally common) non-CBCR case, i.e., a case of a disease not dealt with in the course?

We will also calculate correlations between students’ CBCR course examination results and the outcome measures (illness script richness, correct diagnoses) in our study, to investigate whether students who score better on the test also perform better on the experimental tasks. Separate correlations will be calculated for the CBCR and non-CBCR illnesses to test whether the CBCR course has a general effect that extends to non-CBCR cases or is limited to diseases dealt with in the course.

## Methods

### Study design and participants

We performed a within-subjects experimental study at the University Medical Centre of Utrecht, the Netherlands. Participants were second-year medical students who had participated in the CBCR course and had completed the examination in 2016. Participants were excluded if they had missed six or more out of twelve CBCR sessions or if they had not completed the exam in the first run. A power analysis could not be conducted because we had no information about the size of the expected effect in advance. Students who volunteered to participate received a 10 Euro gift card and a lottery ticket to win an iPad. This study was approved by the Ethical Review Board of the Dutch Association for Medical Education. Informed consent was obtained from all participants.

### Collection of materials

The experiment was conducted in two consecutive sessions. For the first session, a sample of five (of the nine) diseases discussed during the year-2 CBCR course was selected (“CBCR diseases”). This sample was supplemented with ten other diseases of similar complexity and frequency of occurrence that did not receive specific emphasis in the curriculum – though students may have learned about these diseases in a non systematic way, they were not included in any examination in the regular curriculum (we carefully checked this). We called the latter sample of ten diseases “non-CBCR diseases.” The fifteen diseases selected formed the experimental set used in the first session of the experiment (Table 1, left column).

**Table 1:** Illnesses used in the study

Illness script richness^*^	Diagnostic accuracy^*^
Otitis media	Multiple Sclerosis
Meningitis	Parkinson’s disease
ADHD	Gallstones
Atrial fibrillation	Pericarditis
Mamma carcinoma	Vulvovaginal Candidiasis
Polycystic ovarian syndrome	Gonarthrosis
Graves’ disease	Rheumatoid arthritis
Asthma	Hashimoto hypothyroidism
Diverticulitis	*Herniated nucleus pulposus*
Deep vein thrombosis	*Iron deficiency anemia *
*Migraine*	*Benign prostatic hypertrophy*
*Delirium*	*(Non) Hodgkin's lymphoma*
*Uterus myoma *	
*Diabetes Mellitus*	
*Crohn’s disease*	

*Case-Based Clinical Reasoning (CBCR) diseases in italics

The materials for the second session consisted of twelve case descriptions, four of which had been discussed extensively during the year-2 CBCR course (“CBCR cases”). The remaining eight cases were “non-CBCR cases,” i.e., cases of diseases not dealt with in the CBCR course. Students were not familiar with these cases (Table 1, right column).

The case descriptions used in the second session consisted of a verbal description and a photographic portrait of the patient’s face. The patient was typical for the disease (e.g., an obese woman older than 50 years of age for the disease “osteoarthritis of the knee”), and the portrait did not provide any disease-specific information apart from the patients’ age and sex. Typical history and physical examination results were provided in short statements. Table 2 shows a representative example of a case description.

**Table 2:** Example of a case description

History
· Slowly progressive pain in left knee since about one year
· Morning stiffness for less than 30 minutes
· Pain is worse at the end of the day and during walking or bending
Examination
· Bony enlargement left knee
· Tenderness medial joint space
· Crepitus on active motion of the knee
· Limited extension of the knee

The two sessions can be considered independent entities within the experiment in the sense that there was no overlap in the materials used. Within each session, the order of diagnoses (first session) and cases (second session) was randomized in advance, to control for possible sequence effects. Prior to the experiments, the materials were tested with six recently graduated medical students and five final year medical students. This led to the exclusion of one case, being judged as too difficult for our participants.

### Procedure

All participants were tested individually in May 2016, at least one month after they had completed the CBCR examination. Students were not informed that the study contained CBCR illnesses and were told that no specific preparation would be required. Each participant was seated in front of a laptop with a hand-held recording device. The experiment consisted of two consecutive sessions, separated by a five-minute break. Each session was preceded by a few slides providing instructions and a practical example (allergic contact dermatitis). The instruction, experiments, and debriefing took approximately one hour altogether.

At the beginning of the first session, participants were informed that 15 consecutive diseases would be presented and that they were asked to tell everything that came to their mind about each disease. During presentation of the practice example, participants were explicitly reminded of several categories of disease information that they could mention: pathophysiology (e.g., histamine, Langerhans cells), predisposing features (e.g., working with nickel), clinical features (e.g., rash at the side of exposure, itching, vesicles), test results (e. g., positive patch tests), and management (e. g., ointment, topical corticosteroids). During presentation of the experimental diseases, participants were not cued in this way, because we were interested in information they would spontaneously provide upon being cued exclusively with the name of the disease. Two minutes were available for each disease; the pilot study had demonstrated this to be more than sufficient.

In the second session, participants were asked to name the first disease (diagnostic hypothesis) that “popped into their minds” upon reading a case text. The cases were visible for 45 seconds on the screen. The session started with a practice case (allergic contact dermatitis). Subsequently, the twelve experimental cases were presented. Participants were instructed to think aloud, and again the whole procedure was audio-recorded. If any time was left after they had mentioned their first diagnostic hypothesis, participants could use the remaining time to elaborate on a differential diagnosis, if they had other diagnostic options in mind. To prevent them from speculating too much about irrelevant diseases, participants were instructed to treat unmentioned symptoms or findings as “absent” and unmentioned variables as “within the normal range” (e.g., if no mention was made of “fever” in a case, participants were instructed to consider body temperature to be within the normal range).

### Data collection methods

All recorded data were transcribed verbatim by an external transcription service. The amount of information volunteered by the students in the first session was the operationalization of illness script richness. The number of statements was determined by counting the information units provided by the participants, according to a procedure described in previous studies.^5^^,^^17^ Each medically relevant information unit was awarded one point. For example, when a participant said, “this disease is common in middle-aged women and presents with unilateral headaches,” this was counted as five points: one for sex of the patient, one for age, one for complaint (pain), one for organ (head) and one for location (unilateral). In addition, the information was categorized according to the major illness script components: Fault, Enabling Conditions, Consequences, and Management. A fifth component, Miscellaneous, was used to categorize medically relevant information that did not fit into the regular illness script categories (e.g., information about frequency of occurrence). For example, the above statement would award the participant two points in Enabling Conditions (age and sex of the patient) and three points in Consequences (complaint, organ, and location). One of the researchers (YK) performed the coding; inter- and intra-rater reliability (IRR) of the coding was evaluated in a random sample of 10% of the transcripts, coded independently by two of the researchers (YK and SvD).^18^

The total number of statements mentioned was calculated separately for CBCR diseases and non-CBCR diseases, and expressed, for each participant, as the average score over the two categories of diseases (CBCR and non-CBCR), to account for the difference in numbers (five CBCR and ten non-CBCR diseases). These two values were considered to reflect the richness of participants’ illness scripts. Besides, to investigate the maturity of participants’ illness scripts (in addition to richness), proportions of statements for the different components of illness scripts were calculated for each case and again collapsed separately over the five CBCR and ten non-CBCR cases, respectively. Relative contribution of information in each category (Enabling Conditions, Fault, Consequences, Management) adds to the information provided by absolute number, as proportional values “control” for inter-participant differences in wordiness. Otherwise, participants who provide more information would inevitably have more mature illness scripts.

Diagnostic performance was calculated as follows: two points were awarded for each correct diagnosis (e.g., rheumatoid arthritis), and one point for an incomplete or partly correct diagnosis (e.g., arthritis). This is similar to the procedure used by Schmidt et al.^19^ If the correct diagnosis ranked second or further down on the differential, also one point was awarded. Else, no points were awarded. Diagnostic performance scores were also calculated separately for CBCR cases and non-CBCR cases and expressed as averages per case type to account of the difference in numbers (four CBCR cases and eight non-CBCR cases).

Finally, participants’ scores on both parts of the experiment were correlated with their CBCR course final examination scores. This examination consisted of two parts, which students sat at different occasions (in December 2015 and April 2016) and contained 43 items and 50 items, respectively. The results of both examinations were combined into a single score for each participant, expressed on the Dutch “grading scale,” which ranges from 1 (worst) to 10 (best possible performance).

### Data analysis

SPSS version 21 was used for statistical analyses. The differences in participants’ illness script richness and maturity between CBCR and non-CBCR diseases and the differences in diagnostic performance between CBCR and non-CBCR cases were tested for significance using paired t-tests, CBCR versus non-CBCR being the within-subjects independent variable. Significance for all tests was set at p<0.05. Effect sizes were computed according to Cohen’s d (≤ 0.2 representing a small-size effect, 0.2–0.5 representing a medium-size effect, ≥ 0.8 representing a large-size effect).^20^ The association between participants’ CBCR test results and their illness script richness and diagnostic performance was calculated using Pearson’s correlation coefficient r.

## Results

In the academic year 2015-2016, 305 medical students completed the CBCR course at the University Medical Centre at Utrecht. Of these students, 32 (24 female and 8 male students) volunteered to participate in the current study. Most of these students attended all sessions of the course, four students missed one session, and one student missed two (out of nine) sessions. The mean final mark for the CBCR course of the study population was 7.35 (SD=0.74) on the grading scale from 1–10. The 273 students who did not participate had a mean mark of 7.29 (SD=0.65). Thus, in terms of these test scores, the study population was representative of the full population.

### Illness script richness

Table 3 summarizes the illness script richness scores of the participants across the two conditions. For the 15 illnesses included in the analysis, participants mentioned a mean number of 12.91 (SD=2.89) statements per illness. Paired t-tests revealed a significant difference between the illness script richness for CBCR illnesses (M=14.47, SD=3.25) versus non-CBCR illnesses (M=12.145 SD=2.80) (t= 9.47 df=31, p<0.001). Participants mentioned approximately 20% more statements for CBCR diseases than for non-CBCR diseases. The Cohen’s d effect size of the difference was close to large (0.77).20

### Illness script maturity

Table 3 also shows the proportion of statements in each illness script category, which reveals illness script maturity. For both the CBCR and the non-CBCR illnesses, most statements were mentioned in the category Consequences (i.e., signs and symptoms). However, for the CBCR illnesses, this was significantly more prominent than for the non-CBCR illnesses (36.15% (SD=6.75) versus 27.62% (SD=5.65), t=7.20, df=31, p<0.001, Cohen’s d 1.37). The second largest category of statements for both groups was the Fault (i.e., knowledge of pathophysiology). Of all statements of the CBCR illnesses 21.82% (SD=6.36) was related to Fault while 26.56% (SD=6.15) of the statements about the non-CBCR illnesses were categorized as Fault (t=3.72, df=32, p<0.005, Cohen’s d = .76). Similarly, the relative contribution of knowledge of Enabling Conditions was also larger for CBCR diseases (16.05%, SD=5.89) than for non-CBCR diseases (12.45%, SD=4.04).

### Diagnosis of cases

In the second session, the average number of points received for each correct diagnosis was 1.31 (SD=0.24). Diagnostic performance on CBCR cases was significantly better than on non-CBCR cases, 1.63 (SD=0.32) and 1.15 (SD=0.29) points on the average, respectively (t=7.47, df=31, p<0.001, Cohen’s d =1.58).

In addition, we found a marginally significant correlation between students’ illness script richness and their diagnostic accuracy over the two sets of cases (CBCR and non-CBCR) combined (r=0.354, p<0.05). This suggests there is a small relationship between students’ illness script richness (how much they know) and their diagnostic performance (how well they can diagnose cases).

**Table 3:** Illness script richness and structure for CBCR and non-CBCR illnesses (N=32)

Illness script	CBCR diseases^*^	non-CBCR diseases^†^	p	Cohen’s *d*
	Mean% (SD)	Mean% (SD)		
Total statements^‡^	14.47 (3.25)	12.14 (2.80)	0.000	0.768
Enabling Conditions^§^	16.05 (5.89)	12.45 (4.04)	0.001	0.715
Fault^§^	21.82 (6.36)	26.56 (6.15)	0.001	-0.757
Consequences^§^	36.15 (6.75)	27.62 (5.65)	0.000	1.370
Management^§^	18.70 (5.66)	20.99 (4.39)	0.024	-0.452
Miscellaneous^§^	7.28 (4.04)	12.39 (3.86)	0.000	-1.295

*Discussed during CBCR sessions (n=5); †Not discussed during CBCR sessions (n=10); ‡Illness script richness: mean number of statements per illness; §Proportion of the total number of statements mentioned.

### Relationship with CBCR examination results

Pearson’s correlation coefficient r revealed no statistically significant correlation between the CBCR examination results and illness script richness, i.e., neither for the number of statements mentioned for the CBCR diseases (r = –0.006; p=NS), nor for the number of statements mentioned for the non-CBCR diseases (r = –0.176, p=NS).

A significant correlation between CBCR examination results and diagnostic accuracy on CBCR cases was found (r = 0.571, p<0.005). No correlation worth speaking of was found between the CBCR examination results and diagnostic accuracy in non-CBCR cases (r=0.094, p=NS).

## Discussion

### Illness script richness and maturity

Our study shows that one month after completing the CBCR course, students have richer illness scripts of diseases systematically discussed in CBCR sessions than of similarly common diseases that receive less emphasis in the undergraduate curriculum. Moreover, diagnostic performance on CBCR cases is superior to diagnostic performance on cases of similar non-CBCR diseases. Our findings underscore the relevance of a CBCR course for the development of clinical reasoning skills, expressed here as richer illness scripts, and better diagnostic performance. These are encouraging results, as previous literature has primarily stressed the need for real-life patient contact to develop and refine illness scripts.^8^^,^^21^ Our results show that even before they have received any practical clinical training, students benefit from a course in clinical reasoning. In this way, a CBCR course can form a bridge for novices to their first clinical experience.

Not only the richness of illness scripts changes with experience but also their maturity, i.e., their structure and composition.^5^ For experienced physicians, the biomedical details of the Fault (the pathophysiology of the disease) are of little value in the diagnostic process because they rely in many cases on pattern recognition. For novices, on the other hand, who have seen few, if any, patients with a particular disease, knowledge of the Fault plays a more prominent role in their diagnostic reasoning.^5^^,^^22^^-^^24^ Though much of the knowledge volunteered by our second-year medical students revolves around the Fault – they are still inexperienced – the relative contribution of this knowledge was lower for CBCR cases than for non-CBCR cases. Similarly, we also see a shift of the script structure towards a greater role of Enabling Conditions in our study population. This means that patient-oriented information has become more prominent after extended exposure to the diseases in our CBCR course. Though these results should be treated with some caution – proportions add to 1.0 and cannot increase or decrease independently from each other – this finding of increased importance of Enabling Conditions is also in line with earlier findings.^5^^,^^10^ The repeated application of knowledge in the CBCR sessions probably has already tuned our participants’ illness scripts of the discussed diseases toward future use in practical situations.

Overall, students who have richer illness scripts tend to show better diagnostic performance on cases of diseases dealt with in the CBCR course as well as on cases of other diseases; this relationship (r = 0.35) is indicative of approximately 10% common variance. Illness script theory does not consider script richness itself necessary for superior diagnostic performance – students who learn a clinical textbook by heart may not become better diagnosticians. In addition, it would not explain the relationship between CBCR course examination results and students’ script richness of scripts for non-CBCR diseases. Rather, in our view, it suggests a “general” student effect (some students are just “better” in many respects than other students: they have more knowledge of diseases and are better diagnosticians).

### CBCR examination performance

CBCR examination results have predictive value for diagnostic performance on CBCR cases, but not for non-CBCR cases. This suggests students do not develop a more general clinical reasoning ability, which confirms the belief that the development of clinical reasoning skills is disease specific.^11^^,^^25^ This is also what would be predicted on basis of the model by Rumelhart and Norman;^9^ that is, it is hard to see how tuning and restructuring of disease-specific knowledge could influence the development of an illness script for a different disease, unless the diseases share a common denominator, which is usually not the case for different diseases.

Contrary to our expectations, we did not find a correlation between students’ CBCR examination results and their illness script richness. One explanation for this could be the nature of the CBCR examination, which consists solely of closed-format questions of the extended matching type and hence, capitalizes on recognition – the examination does not require students to recall or otherwise generate disease knowledge. In contrast, the first session of our experiment, in which we assessed illness script richness, does draw upon students’ ability to recall or generate this information. In addition, there may be some inherent instability in our measure of illness script richness, partly as a consequence of marginal knowledge (knowledge that cannot be consistently recalled),^26^ partly as a consequence of interpersonal differences in wordiness.

Our findings can be easily aligned with the accretion-restructuring-tuning view of learning.^9^ While in general accretion will be the primary process early in the curriculum and restructuring and tuning later in the curriculum, the CBCR course does appear to contribute to early restructuring of knowledge acquired in previous blocks (a process of accretion), to serve the development of coherent illness scripts. Our finding of a higher diagnostic accuracy for cases studied in the CBCR course suggests tuning, i.e. making knowledge accessible in a context in which it is relevant, is also at work.

### Limitations of the study

First, the number of participants in our study was limited, which may have affected the internal validity of the study. However, this limitation is mitigated by our within-subjects design, which is less vulnerable than the more common between-subjects (control group) design. Next, it is unclear to what extent our results can be generalized to students who did not participate in the study. In any case, we did not find a “volunteer effect”, i.e., that the students who participated were across the board better than the cohort as a whole.^27^^,^^28^ We only found a marginal difference in CBCR examination score (7.35 for students who participated versus 7.29 for students who did not). There may be, however, other differences not captured by course results that limit external generalization.

Another limitation of our study is that we did not directly compare different approaches to teaching clinical reasoning and hence cannot say whether the CBCR method is superior to any other approach. The mandatory nature of our CBCR course precludes a direct comparison with other teaching formats. There is some indirect evidence, though. Schmidt et al. compared students’ diagnostic performance on fairly common cases at three different Dutch universities: one PBL school, one school with a conventional curriculum, and one school with an integrated curriculum.^19^ In Year 2 and 3, the students at this latter school – Amsterdam University Medical School – outperformed students at the two other schools. And these were exactly the cohorts which, at the Amsterdam University Medical School, had attended the predecessor of our current CBCR course. This course might have been responsible for their superior performance, compared with that of students at the two other schools.

Finally, one could argue that the performance of our students may be a general effect of the effort students had invested in learning the CBCR cases, rather than a result of any specific teaching format.^29^ While in general it may be assumed that if students invest more effort, they will also learn more, the CBCR format appears to be more appealing to students than other formats, in particular, traditional formats that rely heavily on lectures, seminars, or textbooks. Working through a whole case that is, if not “authentic”, at least representative of many students’ future practice is experienced as stimulating and discussing possible diagnoses may help students in developing their clinical reasoning. Through CBCR, students experience that discussing and solving cases may be exciting and they are not rewarded for “jumping to conclusions” as will often be the case in a busy practice where they are working under time pressure.

## Conclusions

A CBCR-course like the one included in the medical Bachelor-curriculum at the University Medical Center at Utrecht, the Netherlands, has a positive effect on the development of students’ illness script richness, illness script maturity, and diagnostic performance. It appears the course helps students in restructuring and tuning their clinical knowledge, to make it better available for use in practice. We cannot conclude, however, that a CBCR course is the only way to achieve this and our study also shows that such a course does not necessarily improve students’ general clinical reasoning ability. We doubt, however, whether there is any other educational intervention that would achieve this latter aim.

### Acknowledgements

The authors thank the students who participated in the study

### Conflict of Interest

The authors declare that they have no conflict of interest.
